# On the Inference of Functional Circadian Networks Using Granger Causality

**DOI:** 10.1371/journal.pone.0137540

**Published:** 2015-09-28

**Authors:** Arya Pourzanjani, Erik D. Herzog, Linda R. Petzold

**Affiliations:** 1 Department of Computer Science, University of California, Santa Barbara, Santa Barbara, California, United States of America; 2 Department of Biology, Washington University, St. Louis, Missouri, United States of America; Universiteit Gent, BELGIUM

## Abstract

Being able to infer one way direct connections in an oscillatory network such as the suprachiastmatic nucleus (SCN) of the mammalian brain using time series data is difficult but crucial to understanding network dynamics. Although techniques have been developed for inferring networks from time series data, there have been no attempts to adapt these techniques to infer directional connections in oscillatory time series, while accurately distinguishing between direct and indirect connections. In this paper an adaptation of Granger Causality is proposed that allows for inference of circadian networks and oscillatory networks in general called Adaptive Frequency Granger Causality (AFGC). Additionally, an extension of this method is proposed to infer networks with large numbers of cells called LASSO AFGC. The method was validated using simulated data from several different networks. For the smaller networks the method was able to identify all one way direct connections without identifying connections that were not present. For larger networks of up to twenty cells the method shows excellent performance in identifying true and false connections; this is quantified by an area-under-the-curve (AUC) 96.88%. We note that this method like other Granger Causality-based methods, is based on the detection of high frequency signals propagating between cell traces. Thus it requires a relatively high sampling rate and a network that can propagate high frequency signals.

## Introduction

To understand how complex behaviors arise, we must learn how populations of elements communicate with each other to produce coherent outputs. For example, in the brain, neurons dynamically interact with each other to represent, store and respond to the physical world in real time. Many methods have been developed to discriminate and map functional connections [1–5]. In some cases, one method works well to identify connections at one time scale, but fails to map interactions that occur on other time scales. For example, we recently developed Between-Sample Analysis of Connectivity (BSAC) to map functional GABA connections within a network of circadian neurons [6]. Although BSAC could discriminate the relatively fast and weak excitatory and inhibitory interactions between hundreds of neurons with a high hit rate and low false alarm rate, it did not identify the slower interactions that synchronize circadian rhythms among cells. This highlights the need for different approaches to identify functional connections that may differ in their topologies (e.g. the number or strength of incoming or outgoing connections per node) or dynamics (e.g. when and for how long nodes interact) within the same network of elements.

Synchrony among circadian cells is essential for daily rhythms in physiology and behaviors including sleep-wake, hormone release and metabolism. The suprachiasmatic nucleus (SCN) of the hypothalamus is comprised of a population of approximately 20,000, intrinsically circadian, cells that synchronize their daily rhythms to each other. This is an excellent system for developing algorithms for mapping functional connectivity. Enough is known about how rhythms are generated and synchronized that we can use computational methods to simulate the network. The population of cells is small enough that we can compute their performance over time. There is strong interest in identifying the network topology within the SCN, as disruptions in the network may underlie diverse behaviors from seasonal reproduction to jetlag to fragmented sleep in aging [7–11].

Although approaches for inferring network connections from spike train data [12, 13] and simple oscillators exist [14–19], adapting these methods to infer functional connections in the SCN from gene expression data is difficult. The characteristically unique and fast nature of spike train data make these techniques difficult to apply to circadian gene expression data. Similarly, inference techniques for networks of simple oscillators have not been successfully applied to gene expression data of circadian systems as they are highly complex compared to simple oscillators such as the Kuramoto oscillator.

**Fig 1:**
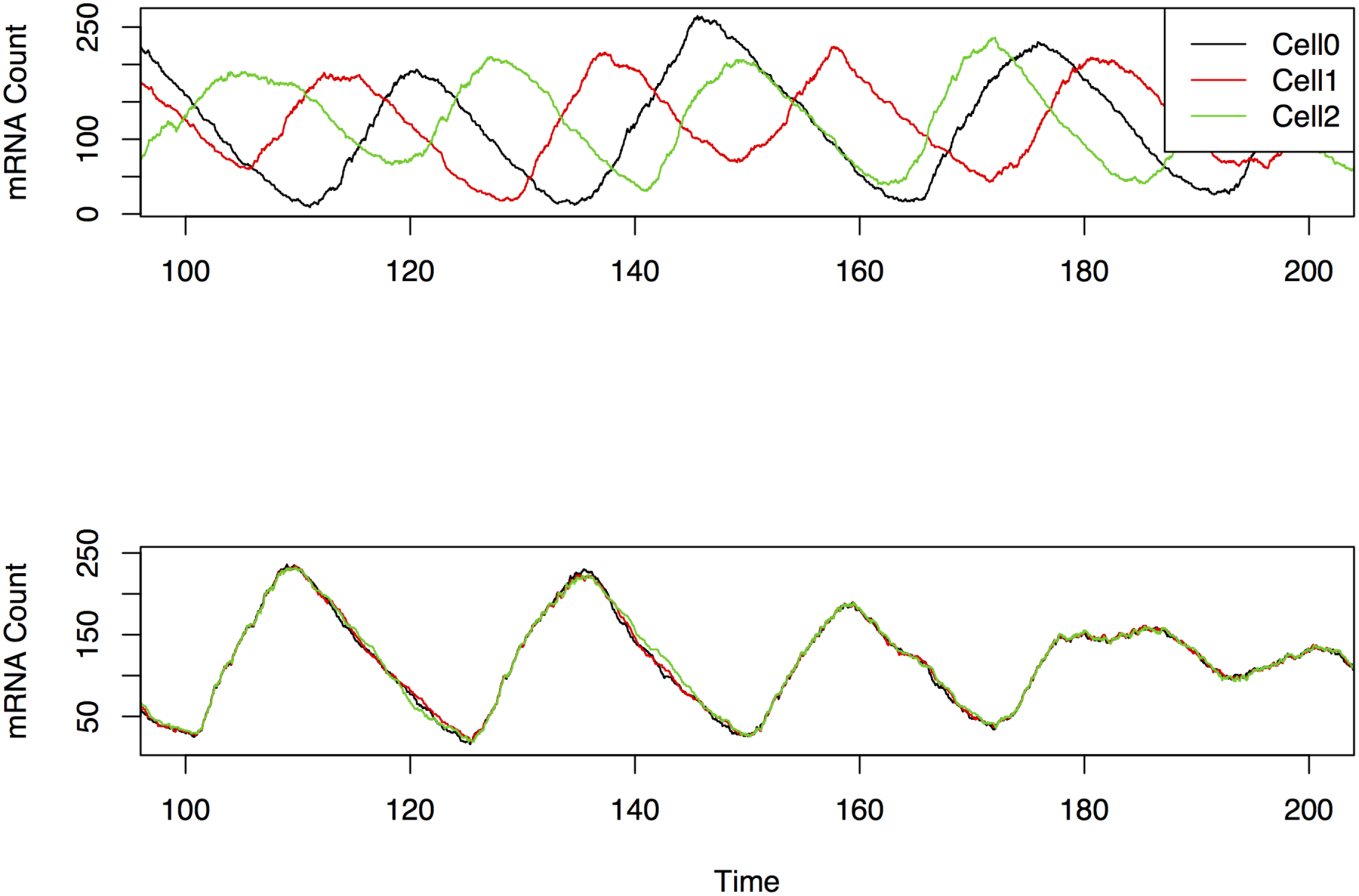
An example of computer simulated PER mRNA cell traces for three different cells in an uncoupled setting (upper plot) and coupled setting (lower plot). Although the difference is visually obvious, it is nearly impossible from the raw traces to tell the directionality of influence between cells and whether connections are direct or indirect.

Although there is a clear difference in appearance between synchronized and unsynchronized raw cell traces (see Fig 1), it is very difficult to tell from these raw traces of gene expression the directionality of influence between cells. Even if the directionality of influence is identified, it is difficult to tell when a cell is directly influencing another cell or simply influencing that cell via a mediator cell (indirect connection).

Traditionally, Granger Causality has been a popular choice for inference of networks in general [20–22]. However, Granger Causality is highly reliant on the assumption that the time series are stationary [23]. The highly non stationary nature of gene expression data from circadian systems is widely known [24, 25] and makes Granger Causality a poor choice for analyzing networks of circadian oscillators using gene expression data. Classical time series techniques for obtaining stationary time series from seasonal data, i.e. data with a repeating or cyclical pattern, rely on obtaining the dominant period of the time series and applying seasonal differencing [26]. While techniques exists for obtaining this dominant period from circadian gene expression data [27], these techniques assume a single stationary period for the time series, which circadian gene expression data has been shown not to have [24].

We have developed a technique for the inference of functional networks which is based on Granger Causality and tailored specifically to nonlinear oscillatory systems such as the circadian system, which exhibit non constant frequency. The technique, called Adaptive Frequency Granger Causality, or AFGC, involves a series of manipulations that make circadian gene expression data a viable candidate for Granger Causality. Unlike Granger Causality based techniques for spike train data, AFGC does not require stationary data, making it suitable for not only spike train data, but oscillatory data as well. This was previously not possible in the literature.

We test our method on known networks of circadian cells modeled with a stochastic version of the LeLoup and Goldbeter model [28] that is coupled via the mechanism described in [29]. The proposed technique based on Granger Causality was able to identify direct one-way connections between neurons in simulated networks with a high degree of accuracy. Additionally, we show how to use a LASSO Granger based statistical technique, LASSO AFGC, to derive causal relationships when the number of time course observations are small relative to the number of cells in a system.

## Materials and Methods

### Overview of Granger Causality and Its Assumptions

Granger Causality is a statistical technique that has been used successfully in the past for inferring gene networks via time course data [30]. Granger Causality involves modeling multiple time series as a linear system of steady-state (stationary) time series with Gaussian noise then finding the correlation between time series to assess how much they affect each other. Thus the assumption that must hold to reliably conduct Granger Causality analysis is that the value of a time series at a single time is a linear combination of the other time series at previous times plus a Gaussian noise term. Complicated oscillatory networks such as the circadian network are unfortunately not linear and thus do not meet this assumption. We more formally define this assumption in Section 2.4. We show however, how to overcome this limitation and apply AFGC to infer oscillatory networks from nonlinear time series data.

### Overview of Circadian Model and Coupling Mechanism

To derive a unique and fundamentally sound methodology for utilizing Granger Causality for inference of circadian networks, we analyzed a stochastic variant of the LeLoup and Goldbeter circadian cell model, which has been shown to accurately capture characteristics of circadian gene expression data [28]. We chose this model because of its simplicity. The model represents each cell as a three state system consisting of PER mRNA (*M*), cytoplasmic PER protein (*P*_*C*_), and nuclear PER protein (*P*_*N*_). The relationships between these expression levels are given for the *i*th cell by,

$$\begin{array}{ccc} \frac{dM^{\left(i\right)}}{dt} & = & v_{s}^{\left(i\right)}\frac{K_{1}^{n}}{K_{1}^{n}+{\left(P_{N}^{\left(i\right)}\right)}^{n}}-v_{m}\frac{M^{\left(i\right)}}{K_{m}+M^{\left(i\right)}}\\ \frac{dP_{C}^{\left(i\right)}}{dt} & = & k_{s}M^{\left(i\right)}-v_{d}\frac{P_{C}^{\left(i\right)}}{K_{d}+P_{C}^{\left(i\right)}}-k_{1}P_{C}^{\left(i\right)}+k_{2}P_{N}^{\left(i\right)}\\ \frac{dP_{N}^{\left(i\right)}}{dt} & = & k_{1}P_{C}^{\left(i\right)}-k_{2}P_{N}^{\left(i\right)} \end{array}$$ (1)

where *k*_1_, *k*_2_, *K*_*s*_, *v*_*d*_, *v*_*m*_, *K*_1_, and *K*_*d*_ are constants. The coupling between cells was modeled as in [29] via a change in PER expression given by.

$$v_{s}^{\left(i\right)}≔0.83+L+\alpha \left({\bar{M}}^{\left(i\right)}-M^{\left(i\right)}\right),$$ (2)

where ${\bar{M}}^{\left(i\right)}$ is the average PER mRNA level of all cells which directly influence cell *i*, and *α* is a constant that determines the strength of the coupling. Throughout all analysis and simulations, initial conditions and parameters of the model were chosen such that during the interval of data collection cells were in approximate synchrony. We converted the ordinary differential equation model to a discrete stochastic model in the usual way, by replacing all concentrations in (Eq 1) by discrete populations. This requires multiplication of the concentrations by a system volume Ω. This system volume controls how closely the stochastic system is to its deterministic counterpart. We chose Ω = 50 in our experiments, as this value was found to best mimic the stochastic properties of cell traces from experimental data such as those found in [31]. To differentiate between the differential equation Model (1) and the stochastic analog, we label all constants and populations with a subscript Ω when working with the discrete stochastic model.

### Granger Causality Applied to Circadian Systems

Our first goal was to detrend the data so that Granger Causality would be applicable. We start by drawing our attention not to *M* ^(*i*)^[*t*] per say, but to *D* ^(*i*)^[*t*]: = *M* ^(*i*)^[*t*] − *M* ^(*i*)^[*t* − 1], i.e. the change in PER mRNA levels of each cell at each instant in time. *D* ^(*i*)^[*t*] is also referred to as the “differenced” version of *M* ^(*i*)^[*t*]. Analyzing the differenced series is useful because when *M* ^(*i*)^[*t*] is approximately linear, its difference, *D* ^(*i*)^[*t*], is stationary and thus suitable for Granger causality inference.

**Fig 2:**
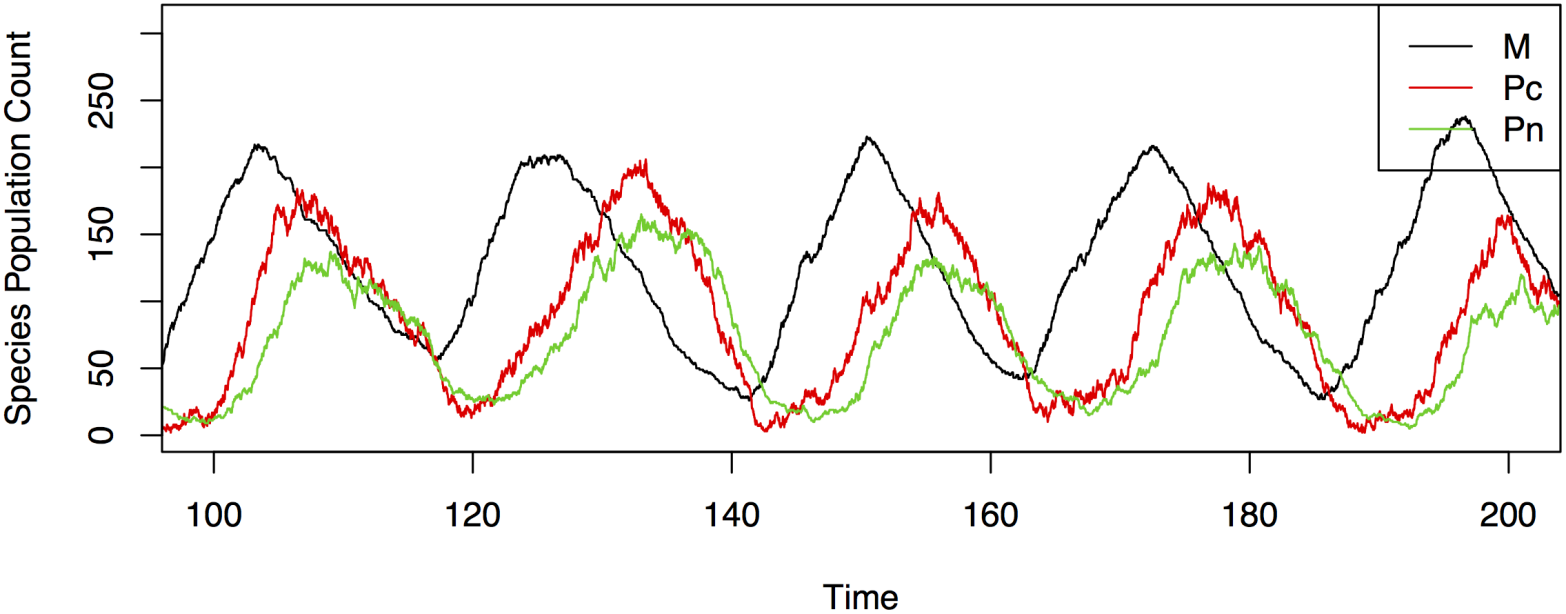
An example of trajectories of species in a single cell over 200 hours. The cell is modeled by the discrete stochastic analogue of the Model (1) with Ω = 50. *M* represents the mRNA levels of the Period gene, *P*_*c*_ represents the cytoplasmic levels of the PERIOD protein, and *P*_*n*_, the nuclear levels of the PERIOD protein. In the model, Period mRNA is translated into PERIOD protein and PERIOD protein feeds back to repress production of Period mRNA. Note that in the subintervals where *M* is increasing, the trajectories of *M* are nearly linear. Additionally, during these subintervals *P*_*N*_ tends to remain nearly constant.

From the theory of Markov processes, we know that in a short period of time *τ*, the change in PER mRNA level is the difference of two Poisson random variables with propensities that are functions of the system at that moment in time, that is

$$\begin{array}{ccc} D_{\Omega }^{\left(i\right)}\left[t+\tau \right] & = & \text{Pois}\;\{\tau v_{s,\Omega }^{\left(i\right)}\left[t\right]\frac{K_{1,\Omega }^{n}}{K_{1,\Omega }^{n}+{\left(P_{N,\Omega }^{\left(i\right)}\left[t\right]\right)}^{n}}\}-\text{Pois}\;\{\tau v_{m,\Omega }\frac{M_{\Omega }^{\left(i\right)}\left[t\right]}{K_{m,\Omega }+M_{\Omega }^{\left(i\right)}\left[t\right]}\}, \end{array}$$ (3)

where $v_{s,\Omega }^{\left(i\right)}\left[t\right]=\Omega v_{s}^{\left(i\right)}$ and *v*_*m*,Ω_ = Ω*v*_*m*_. For now we draw our attention to intervals of the time where PER mRNA is increasing. During these intervals, the system as described by (Eq 1) exhibits several important properties that facilitate the use of Granger Causality:

1. Coupling is more highly expressed in the system. This can be seen from the first equation in (Eq 1). During periods where PER mRNA is increasing, the first term (birth term) is in general much larger than the second term (death term). Since coupling is facilitated through the $v_{s}^{\left(i\right)}$ coefficient of the first term, cells will be more responsive to the signals and fluctuations of other cells during periods of PER mRNA increase. Although other parts of the differenced time series may exhibit properties necessary for Granger Causality (i.e. stationarity and whiteness), the effective low coupling during these periods makes them an unsuitable candidate for Granger Causality since it works best when time series are more sensitive or responsive to the other time series that influence them.
2. $M_{\Omega }^{\left(i\right)}\left[t\right]$ is approximately linear. This can be seen from plots of cell traces of PER mRNA from simulations such as Fig 2. When PER mRNA is increasing, the cell traces appear approximately linear in shape. This allows the data to better fit the linearity mold that Granger Causality (VAR) models require.
3. $P_{N,\Omega }^{\left(i\right)}\left[t\right]$ remains approximately constant in contrast to intervals where PER mRNA is decreasing and $P_{N,\Omega }^{\left(i\right)}\left[t\right]$ is not constant. We show through derivations that when $P_{N,\Omega }^{\left(i\right)}\left[t\right]$ is constant, the system can be reasonably approximated as linear, and is thus suitable for Granger Causality. Since, $P_{N,\Omega }^{\left(i\right)}\left[t\right]$ remains approximately constant then $K_{1,\Omega }^{n}/\left(K_{\Omega }^{n}+{\left(P_{\Omega }^{\left(i\right)}\left[t\right]\right)}^{n}\right)$ also remains approximately constant. This can be seen qualitatively by observing gene expression from simulations of Model (1). Fig 2 depicts how, in a stochastic simulation of Model (1), *P*_*N*_ remains approximately constant as *M* increases for each individual cell.

**Fig 3:**
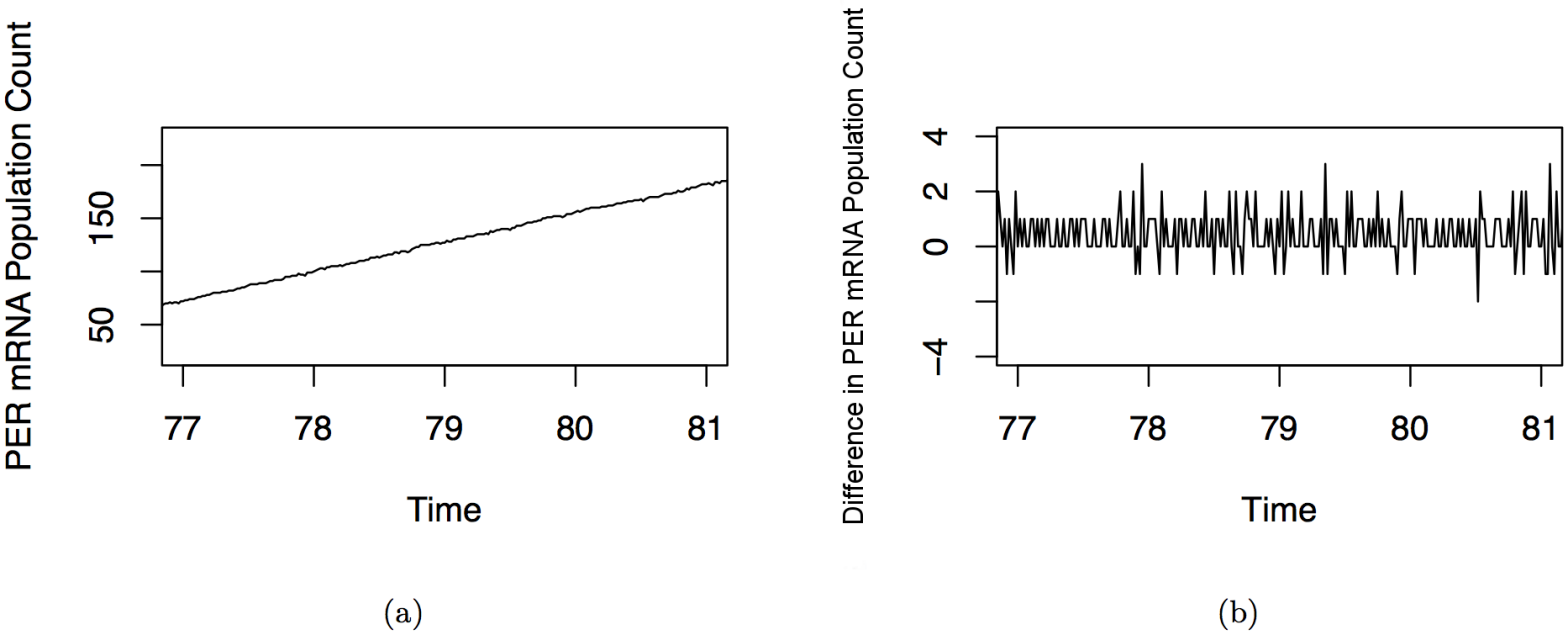
An example of a trajectory of a PER mRNA population level over a four hour period where PER mRNA is increasing. Part A depicts how the PER mRNA population count grows approximately linearly when observed over short periods of time on increasing slopes. Part B is the same time series over the same time period, but differenced over one minute time intervals. Once the approximately linear time series is differenced, the resulting time series is stationary in appearance. The differenced time series can also be interpreted as the change in PER mRNA level at each time point. Although this is only a particular subinterval of the trace, the approximately linear property holds for all subintervals where PER mRNA is increasing. In practice, observations from several of these subintervals (one from each circadian cycle) are used in the network inference method.

**Fig 4:**
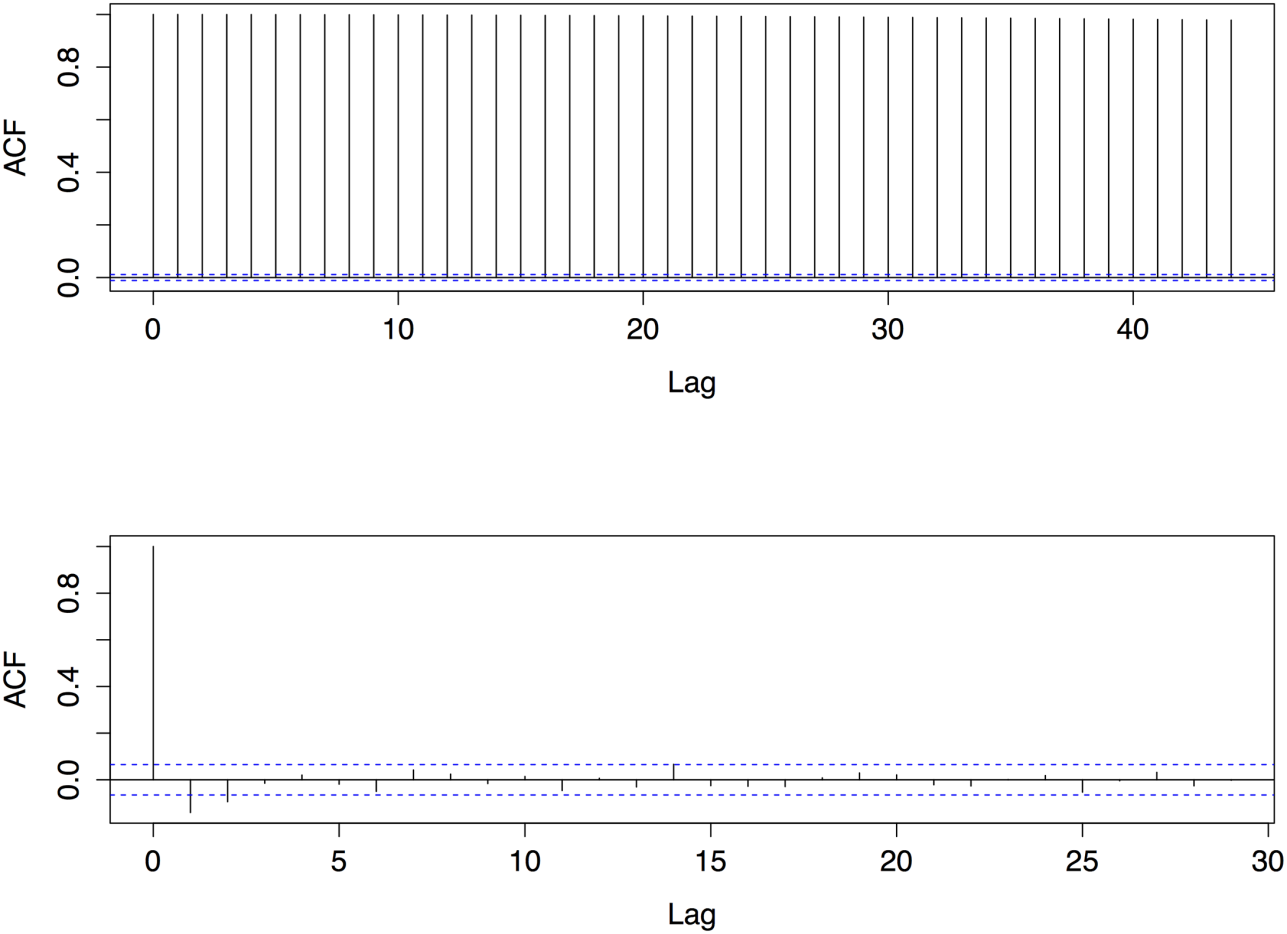
Autocorrelation function of undoctored oscillating PER mRNA sequence (top) and concatenation of differenced increasing PER mRNA segments over the same time period. ACF was calculated on a time series sampled every minute simulated for 500 hours. The concatenated time series is only 15 hours in length as it is derived from the small sub portions of oscillation where PER mRNA is increasing.

From the third property, (Eq 3) can be written as

$$\begin{array}{ccc} D_{\Omega }^{\left(i\right)}\left[t+\tau \right] & \approx & \text{Pois}\;\{\tau c_{\Omega }v_{s,\Omega }^{\left(i\right)}\left[t\right]\}-\text{Pois}\;\{\tau v_{m,\Omega }\frac{M_{\Omega }^{\left(i\right)}\left[t\right]}{K_{m,\Omega }+M_{\Omega }^{\left(i\right)}\left[t\right]}\}, \end{array}$$ (4)

where *c*_Ω_ is a constant. During these small windows where $M_{\Omega }^{\left(i\right)}$ is linear, $D_{\Omega }^{\left(i\right)}$ is stationary. This is illustrated in Fig 3, which shows the $M_{\Omega }^{\left(i\right)}$ trajectory of Fig 2 from time 95 to time 100. Fig 4 shows the difference in the autocorrelation function between an ordinary oscillatory trajectory and a differenced segment of the portion of the time series where mRNA is increasing. The slow drop off in the autocorrelation function for the former is a clear indicator of nonstationarity, while the small spikes at the lags of the latter time ACF indicate a time series that is stationary and can be “whitened”, that the residuals of a VAR model fit to this time series will be white noise.

Continuing with our derivation, expanding the term $v_{s,\Omega }^{\left(i\right)}\left[t\right]$ in the parameter of the first Poisson random variable in (Eq 4) yields

$$\begin{array}{ccc} \tau c_{\Omega }v_{s,\Omega }^{\left(i\right)}\left[t\right] & = & \tau c_{\Omega }\;(0.83\Omega +\alpha \;({\bar{M}}_{\Omega }^{\left(i\right)}\left[t\right]-M_{\Omega }^{\left(i\right)}\left[t\right]))\\ & = & \tau c_{\Omega }\;(0.83\Omega +\alpha \;[(\frac{1}{N}\;\sum_{k}\;M_{\Omega }^{\left(k\right)}\left[t\right])-M_{\Omega }^{\left(i\right)}\left[t\right]])\\ & = & \tau c_{\Omega }\;(0.83\Omega +\alpha \;[\frac{1}{N}\;\sum_{k}\;(M_{\Omega }^{\left(k\right)}\left[t\right]-M_{\Omega }^{\left(i\right)}\left[t\right])]) \end{array}$$ (5)

from which we see that the model coupling was constructed so that $M_{\Omega }^{\left(i\right)}$ is “driven” in time to match the values of the cells it is coupled with. Thus although there are momentary gaps between $M_{\Omega }^{\left(i\right)}$ and $M_{\Omega }^{\left(k\right)}$ due to the stochastic nature of the system, these two quantities will always be near to each other. Furthermore, any momentary difference between the two quantities can be assumed to be due to the last *p* changes in $M_{\Omega }^{\left(k\right)}$, where *p* is some finite integer. We thus make the modeling choice

$$\begin{array}{ccc} \tau c_{\Omega }v_{s,\Omega }^{\left(i\right)}\left[t\right] & = & \tau c_{\Omega }\;(0.83\Omega +\alpha \;[\frac{1}{N}\;\sum_{k}\sum_{j=0}^{p}D_{\Omega }^{\left(k\right)}\left[t\right]])\\ & = & \tau c_{\Omega }\;(0.83\Omega +\sum_{j=0}^{p}\;[\sum_{k}\frac{\alpha }{N}D_{\Omega }^{\left(k\right)}\left[t\right]]). \end{array}$$ (6)

Since the term in the square brackets is simply a linear combination of the $D_{\Omega }^{\left(i\right)}\left[t\right]$, we define a new term $\alpha^{\prime }D_{\Omega }\left[t\right]$ which is the dot product of some constant vector and the difference of mRNA levels in all cells at time *t*, to obtain

$$\tau c_{\Omega }v_{s,\Omega }^{\left(i\right)}\left[t\right]=\tau c_{\Omega }(0.83\Omega +\sum_{j=0}^{p}\alpha^{\prime }D_{\Omega }\left[t\right]),$$ (7)

so that substituting back into (Eq 4) yields

$$\begin{array}{ccc} D_{\Omega }^{\left(i\right)}\left[t+\tau \right] & \approx & \text{Pois}\;\{\tau c_{\Omega }\;(0.83\Omega +\sum_{j=0}^{p}\alpha^{\prime }D_{\Omega }\left[t\right])\}-\text{Pois}\;\{\tau v_{m,\Omega }\frac{M_{\Omega }^{\left(i\right)}\left[t\right]}{K_{m,\Omega }+M_{\Omega }^{\left(i\right)}\left[t\right]}\}\\ & = & \text{Pois}\;\{0.83\Omega \tau c_{\Omega }+\tau c_{\Omega }\sum_{j=0}^{p}\alpha^{\prime }D_{\Omega }\left[t\right]\}-\text{Pois}\;\{\tau v_{m,\Omega }\frac{M_{\Omega }^{\left(i\right)}\left[t\right]}{K_{m,\Omega }+M_{\Omega }^{\left(i\right)}\left[t\right]}\} \end{array}$$ (8)

**Fig 5:**
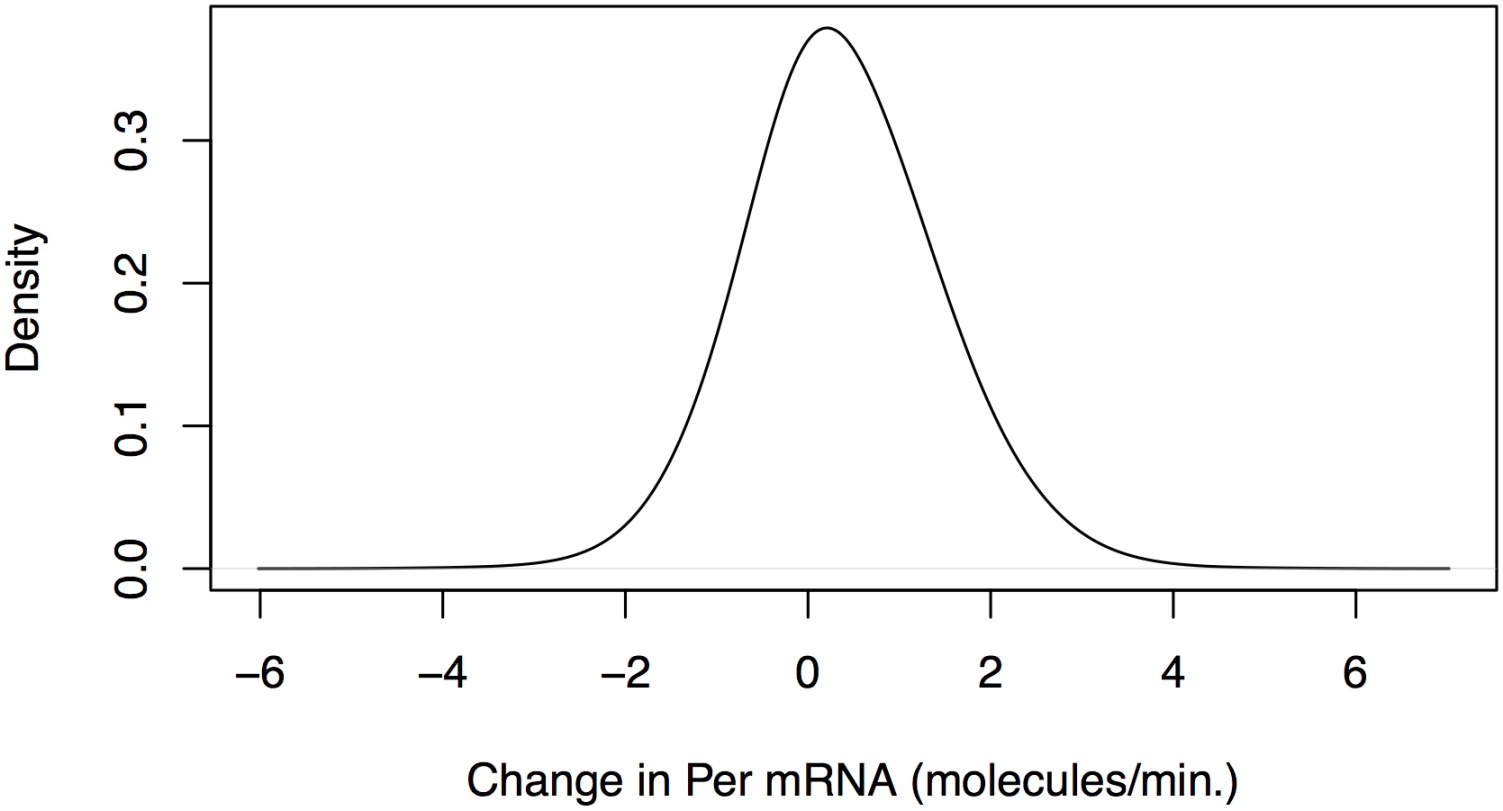
Sample density of the PER mRNA differences, $D_{\omega }^{\left(i\right)}$, from the time series in Fig 3a. Ω = 50 and the samples taken at one minute intervals (*τ* = 1/60). The sample density appears normal, justifying the normality approximation of Eq (8). We see that the mean of the changes in PER mRNA every minute is slightly positive and the range is between -4 and 4, as was the case in Fig 3.

Now we define *λ*_1_ and *λ*_2_ to be the respective propensities of the two Poisson random variables in (Eq 8). If at least one of these quantities is large, which happens when either *τ* is large, Ω is large, or ***α*** is large, then we can use the approximation

$$\begin{array}{ccc} D_{\Omega }^{\left(i\right)}\left[t+\tau \right] & \approx & N\;(\lambda_{1}-\lambda_{2},\lambda_{1}+\lambda_{2})\\ & = & N\;(0.83\Omega \tau c_{\Omega }+\tau c_{\Omega }\sum_{j=0}^{p}\alpha^{\prime }D_{\Omega }\left[t\right]-\lambda_{2},\lambda_{1}+\lambda_{2})\\ & = & 0.83\Omega \tau c_{\Omega }+\tau c_{\Omega }\sum_{j=0}^{p}\alpha^{\prime }D_{\Omega }\left[t\right]-\lambda_{2}+N\;(0,\lambda_{1}+\lambda_{2}) \end{array}$$ (9)

which roughly fits the mold of a vector autogregessive process (VAR) and is thus appropriate for application of Granger Causality. Empirical data from our stochastic model suggests that the normal approximation is appropriate. Fig 5 illustrates a sample density of the changes in PER mRNA, $D_{\Omega }^{\left(i\right)}$, from the time series in Fig 3. Note the characterically Gaussian shape.

We note that although (Eq 9) shows a variance in the random component of our model that is dependent on time, for large enough *λ*_1_ and/or *λ*_2_ the random variable (Eq 8) converges to a normal random variable with constant variance. This constant variance is a necessary assumption of vector autoregressive models and Granger Causality. We found that Ω = 50, *α* = 100, yielded good synchronization.

### Granger Causality and Significance Test

The vector autoregressive (VAR) model is described by

$$y_{t}=\nu +A_{1}y_{t-1}+\cdots +A_{p}y_{t-p}+u_{t},$$ (10)

where **y**_*t*_ represents the state of *K* cells at time *t*, ***ν*** is a constant vector, **A**_1_, ⋯, **A**_*p*_ are constant matrices, and **u**_*t*_ is a vector of normal random variables with mean zero. In scalar form, we write the state of each cell *i* at time *t* as

$$y_{t}^{\left(i\right)}=\nu +\alpha_{i,1}^{\left(1\right)}y_{t-1}^{\left(0\right)}+\cdots +\alpha_{i,K}^{\left(1\right)}y_{t-1}^{\left(K\right)}+\cdots +\alpha_{i,1}^{\left(p\right)}y_{t-p}^{\left(0\right)}+\cdots +\alpha_{i,K}^{\left(p\right)}y_{t-p}^{\left(K\right)}+u_{t}^{\left(i\right)},$$ (11)

where $\alpha_{i,j}^{\left(k\right)}$ is the entry of **A**_*k*_ in the *i*th row and *j*th column. Since (Eq 11) is a linear combination of past states, then by using the noisy past observations of our system, we estimate the coefficients in (Eq 11) by ordinary least squares (OLS) regression. Similarly, we estimate the coefficients in the simpler model

$$\begin{array}{ccc} y_{t}^{\left(i\right)} & = & \nu +\alpha_{i,1}^{\left(1\right)}y_{t-1}^{\left(0\right)}+\cdots +\alpha_{i,j-1}^{\left(1\right)}y_{t-1}^{\left(j-1\right)}+\alpha_{i,j+1}^{\left(1\right)}y_{t-1}^{\left(j+1\right)}+\cdots +\alpha_{i,K}^{\left(1\right)}y_{t-1}^{\left(K\right)}\\ & & +\cdots +\alpha_{i,1}^{\left(p\right)}y_{t-p}^{\left(0\right)}+\cdots +\alpha_{i,j-1}^{\left(1\right)}y_{t-1}^{\left(j-1\right)}+\alpha_{i,j+1}^{\left(1\right)}y_{t-1}^{\left(j+1\right)}+\cdots +\alpha_{i,K}^{\left(p\right)}y_{t-p}^{\left(K\right)}+u_{t}^{\left(i\right)}, \end{array}$$ (12)

which differs from (Eq 11) only in that there is no dependence on cell *j*. From regression estimates we can derive an estimate of standard error ${\hat{\sigma }}_{A}^{2}$ and ${\hat{\sigma }}_{B}^{2}$ for each of these models. These estimators can be interpreted as the amount of variation in $y_{t}^{\left(i\right)}$ that Models (11) and (12) can explain. Clearly the simpler model, (Eq 12), is nested in the Model (11) and thus must explain less of the variation in $y_{t}^{\left(i\right)}$ than Model (11). It can be shown that given our assumptions, these estimators are chi-squared distributed and thus their ratio is *F*-distributed. Under the null hypothesis that cell *i* is not dependent on cell *j*, ${\hat{\sigma }}_{A}^{2}$ and ${\hat{\sigma }}_{B}^{2}$ should be approximately equal, and their ratio, i.e. the *F*-statistic should be close to one. When the *F*-statistic is significantly larger than one, we say that cell *j* Granger Causes cell *i*. The following algorithm shows how this technique can be used to reconstruct the connections of an entire network of cells.

**Algorithm 1:** Algorithm For Reconstructing Functional Circadian Network From Time Series Data

**Data:** K Gene Expression Time Series Corresponding to Each Cell

**Result:** Graph Corresponding to Cell Network

initialize graph *G* = (*V*, *A*) with vertex for each of the *K* cells and no vertices;

**for** *i* = 0 to *K* − 1 **do**

 estimate coefficients ${\hat{\alpha }}_{i,1}^{\left(1\right)},\cdots ,{\hat{\alpha }}_{i,K}^{\left(p\right)}$ for Model (11);

 use these coefficients to estimate standard error of full model, i.e. ${\hat{\sigma }}_{A}^{2}$;

**for** *j* = 0 to *K* − 1 **do**

  estimate coefficients ${\hat{\alpha }}_{i,1}^{\left(1\right)},\cdots ,{\hat{\alpha }}_{i,K}^{\left(p\right)}$ for nested Model (12) not including coefficients for cell *j*;

  use these coefficients to estimate standard error of nested model, i.e. ${\hat{\sigma }}_{B}^{2}$;

  use ${\hat{\sigma }}_{A}^{2}$ and ${\hat{\sigma }}_{B}^{2}$ to calculate *F*-statistic;

**if** *F statistic* > *significance threshold* **then**

   insert directed edge *e* = (*j*, *i*) in to *E*, i.e. cell *j* influences cell *i*;

**end**

**end**

**end**

### Group LASSO Method

**Fig 6:**
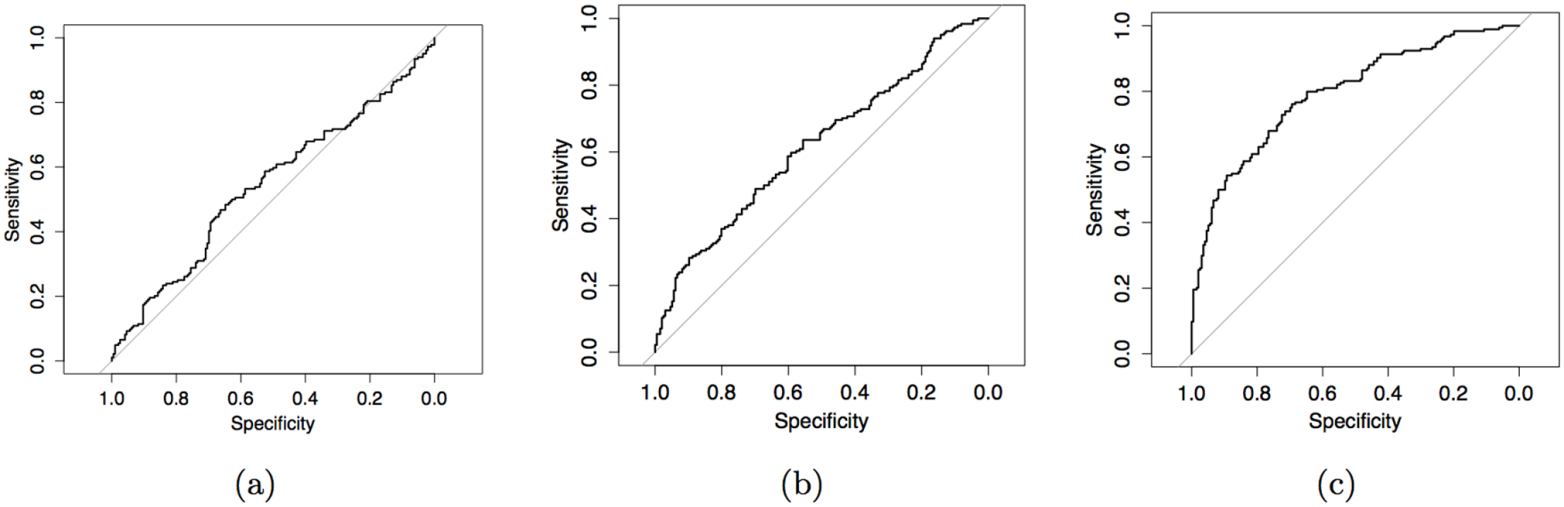
ROC’s of inferred one-way connections for the 10-cell dual chain network in Fig 7. Inferred conducted by AFGC using (a): 6 hours of observations (AUC = 54.35%), (b): 14 hours of observations (AUC = 61.95%), and (c): 28 hours of observations (AUC = 79.33%).

**Fig 7:**
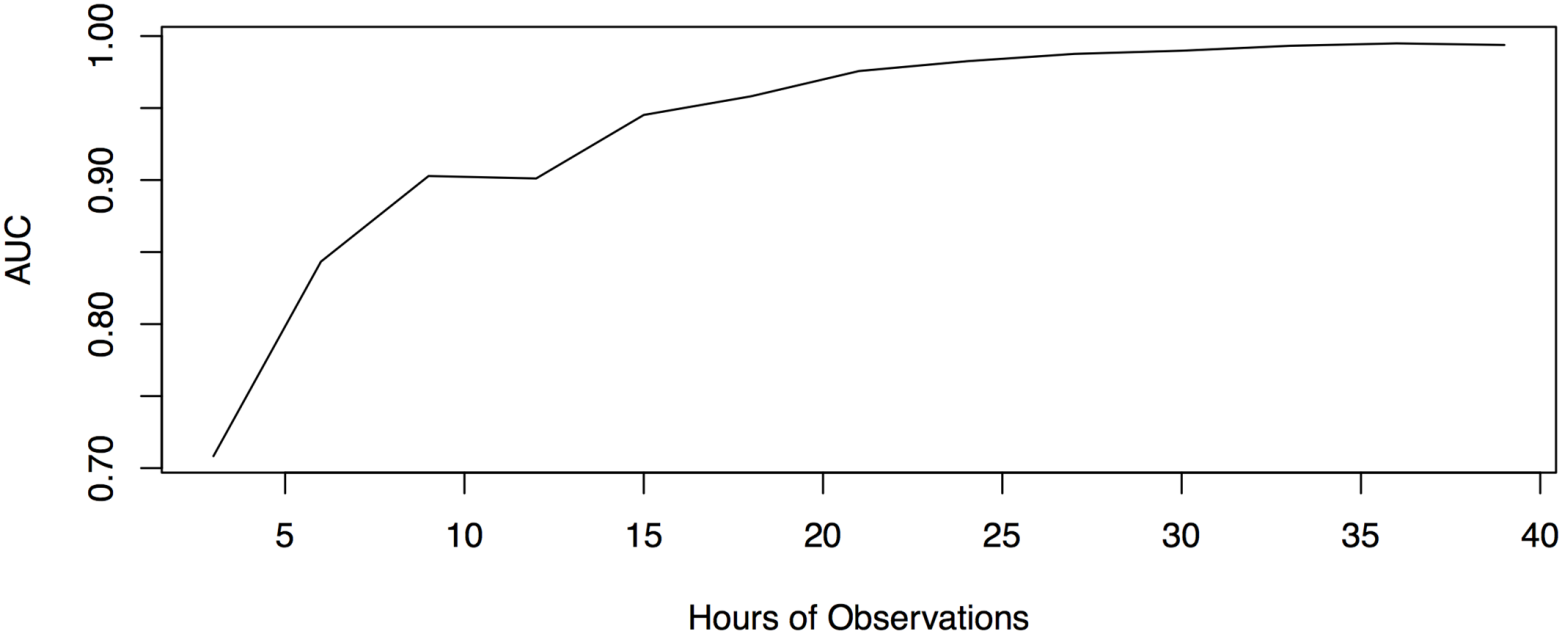
AUC from AFGC as the number of observations in hours increases. Note that observations are only taken from subintervals of the time series where PER mRNA is increasing. Using 40 hours of observations from these intervals (equivalent to about two weeks of total observations) returns an AUC that is nearly equal to one.

For parameter estimation where the number of parameters is large relative to the number of observations, parameter estimates vary highly and can lead to misleading inference. Fig 6 shows receiver operating characteristic curves (ROC) depicting the accuracy of AFGC as the number of observations is increased for a 10 cell model called the 10 cell dual chain network. Here accuracy is defined as the ratio of true-positive connections found to the number of false-positive connections found. In all three plots, the same network is being simulated, with the number of parameters to be estimated given by *Kp* + 1 = 101. As the number of data points is increased, we we see that the area under the ROC curve (AUC) increases and thus the accuracy of our inference if higher, as we would predict. Fig 7 shows how the AUC from the AFGC increases as a function of the number of observations used.

For instances where observations are small relative to the number of parameters being estimated, the group LASSO method [30, 32] has been shown to reduce variance in exchange for bias in parameter estimates, by imposing a penalization on the size of parameter estimates. This lowering of variance often leads to improved results. We propose a modified version of the Granger causality technique with group LASSO in [30] to infer functional cell networks when the number of cells in a system is large relative to the number of observations available for that system. We show in the next section that this method often returns improved results over ordinary Granger Causality inference.

Partition the set of parameters $\left\{\alpha_{i,1}^{\left(1\right)},\cdots ,\alpha_{i,K}^{\left(1\right)},\cdots ,\alpha_{i,1}^{\left(p\right)},\cdots ,\alpha_{i,K}^{\left(p\right)}\right\}$ into *K* groups {G_1_, ⋯, G_*K*_} such that $\alpha_{i,j}^{\left(l\right)}\in G_{k}\Leftrightarrow j=k$. This amounts to grouping all parameters involving the *j*th cell into one group. Let *Y* be the vector of gene expression observations and $\hat{Y}\left(\alpha \right)$ be the estimate of those observations via the estimated parameters in Model (11). The estimates ${\hat{\alpha }}_{Group}$, defined as

$${\hat{\alpha }}_{Group}=\underset{\beta }{\text{argmin}}\;{\left\|Y-\hat{Y}\left(\alpha \right)\right\|}^{2}+\lambda \sum_{k=1}^{K}{\left(\alpha_{i,k}^{\left(1\right)}\right)}^{2}+\cdots +{\left(\alpha_{i,k}^{\left(p\right)}\right)}^{2},$$ (13)

are known as the group LASSO parameter estimates and were first introduced in [30].

For statistical inference, the variance-covariance matrix of the parameter estimates in ${\hat{\alpha }}_{Group}$ must be available. Since there is no known way to calculate this matrix, we approximate this matrix by the variance-covariance matrix of the OLS estimators. Our results demonstrate that this approximation leads to improved accuracy in network inference.

## Results

We demonstrate the effectiveness of our methodology by applying it to the inference of various Circadian networks simulated stochastically. The model we use is a stochastic version of (Eq 1), coupled using the scheme described in [29]. Key parameters in our simulation are the scale concentration to number of molecules, Ω, which we set to 50, and the coupling strength, *α* which we set to 100. Observations of our system are collected once every minute. For larger models where more observations are needed, we concatenate multiple differenced time series from a single realization using of course only the portions of the time series where mRNA is increasing. We then treat this concatenation as a single time series.

**Fig 8:**
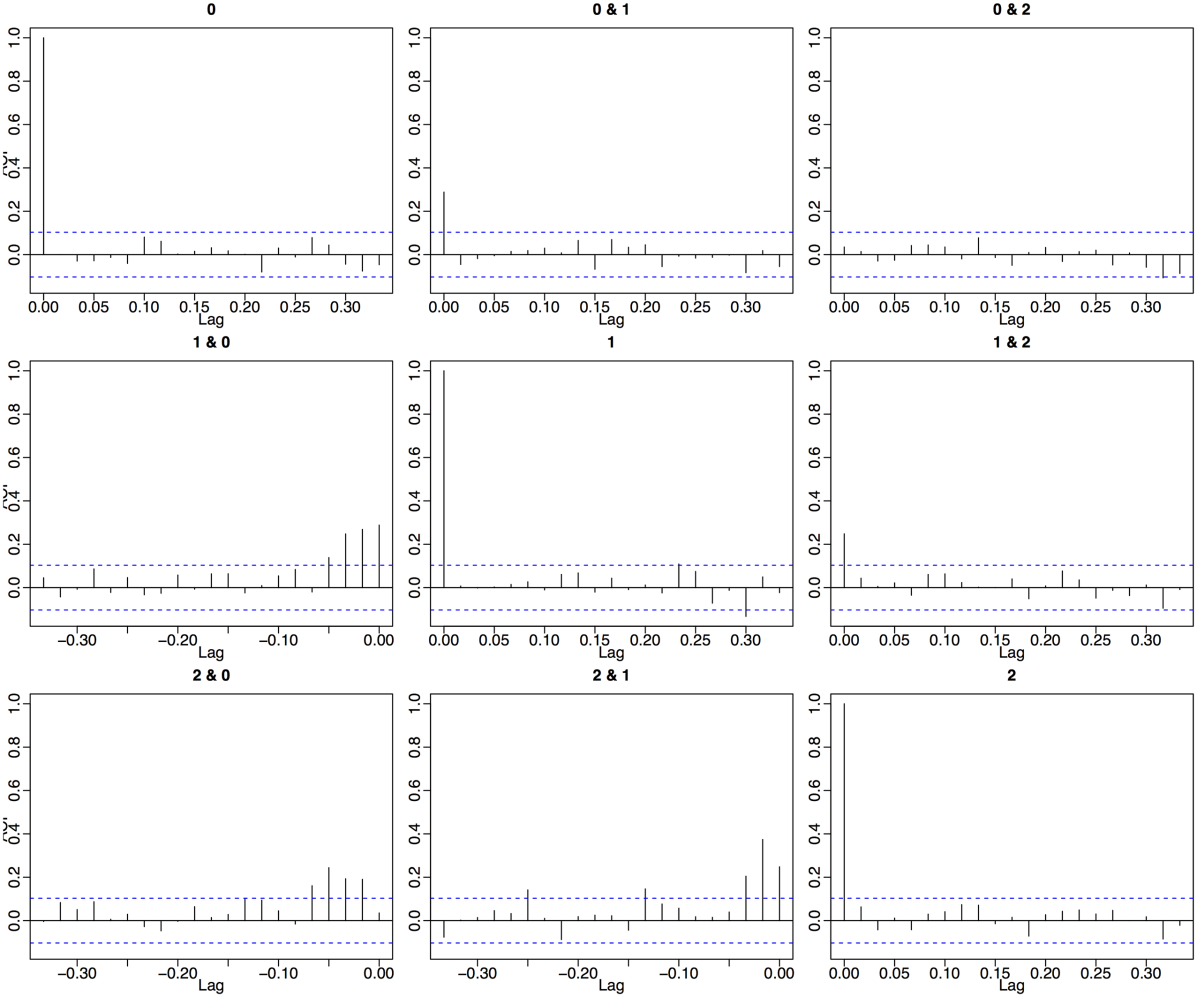
Cross correlation function for differenced realization of 3 cell network. The cross correlation function was calculated using six hours of observations. Significant autocorrelation is not observed while we do see significant cross correlation when cell 0’s time series is lagged against the time series of cell 1 and cell2 and also when cell 1’s time series is lagged against the time series of cell 2.

The model order was chosen to be 5 for all VAR models. This was chosen to reflect the information transfer lag of 5 minutes between cells that we observed empirically in cross correlation functions. Fig 8 is a sample cross correlation function from a realization of the 3 cell network described in the next section. Significant lagged correlations drop off at around 0.07 hours, or about 5 minutes. We also obtained the best network inference results with this choice of model order.

### 3 Cell Network

**Fig 9:**
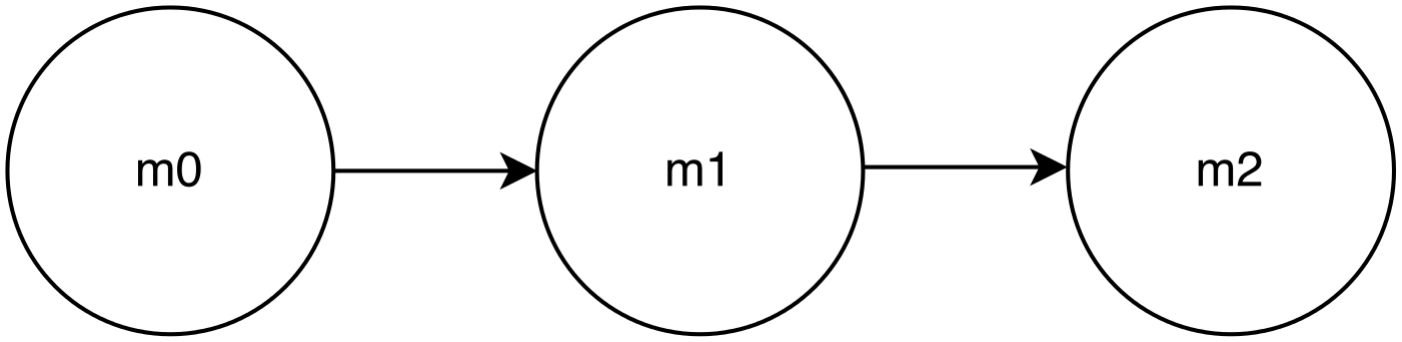
3 Cell Network.

Our first example is the three cell model where all cells are lined up and each cell influences the cell to its right, depicted in Fig 9. We note the indirect connection between cell *m*0 and cell *m*2 in the model. We seek to infer only the direct connections.

Table 1 shows computed *p*-values for the significance of a causal connection from each cell to another. Computation was done on simulated data from the three cell model. Six hours (360 observations) of time series data was used to calculate the statistics.

**Table 1:** *p*-values for one-way connections between cells in 3 cell network.

	To *m*0	To *m*1	To *m*2
From *m*0	NA	0	0.01
From *m*1	0.65	NA	2.2e-16
From *m*2	0.59	0.89	NA

AFGC identifies the true connections (*m*0 to *m*1 and *m*1 to *m*2) with a *p*-value that is at least 15 orders of magnitude greater than the *p*-value assigned to all other connections.

The *p*-values of the true connection (from *m*0 to *m*1 and from *m*1 to *m*2) are several orders of magnitude smaller than the *p*-values of all other connections. Our inference methodology is able to perfectly capture the structure of this network, including not falsely naming the indirect connection as direct. There was no need to use the group LASSO to improve the results.

### 4 Cell Network with Bypass

**Fig 10:**
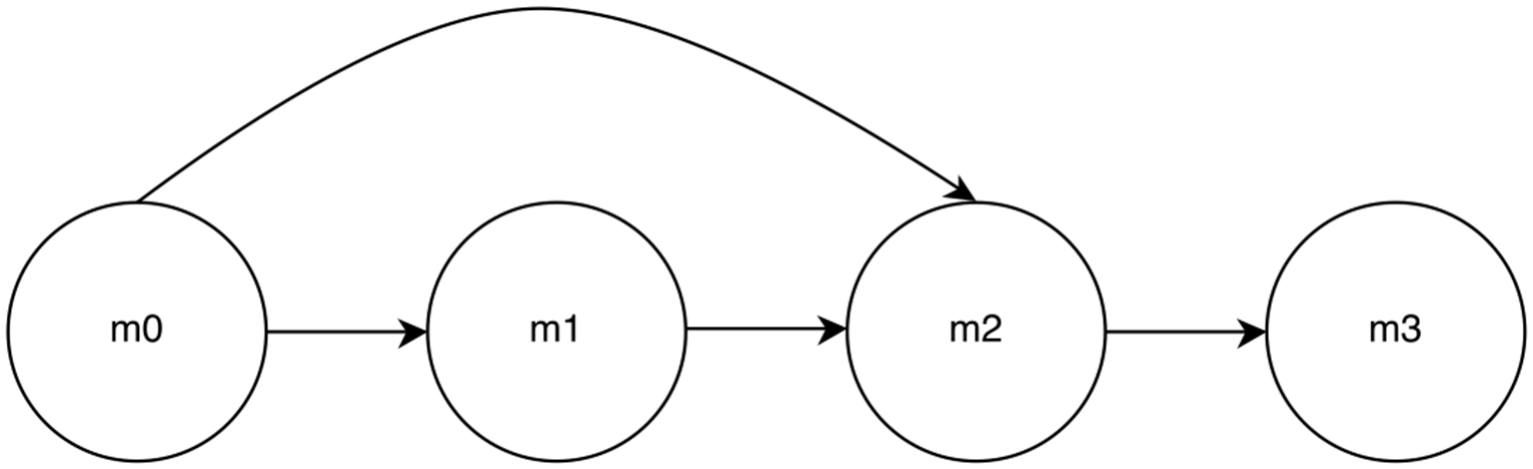
4 Cell Network with Bypass.

To further test our methodology when indirect connections are present, we introduce a 4 cell model with a bypass, depicted in Fig 10.

The *p*-values for inferred connections are shown in Table 2. Seven hours (420 observations) of time series data was used to calculate statistics.

**Table 2:** *p*-values for one-way connections between cells in 4 cell network.

	To *m*0	To *m*1	To *m*2	To *m*3
From *m*0	NA	0	1.53e-10	0.60
From *m*1	0.50	NA	2.75e-06	0.79
From *m*2	0.73	0.01	NA	1.42e-11
From *m*3	0.13	0.01	0.62	NA

AFGC is able to separate true from false connections with an order of magnitude in *p*-value of at least five.

Again, the true connections (*m*0 to *m*1 and *m*2, *m*1 to *m*2, and *m*2 to *m*3) are found to have significance values several orders of magnitude lower than other connections. One again, there was no need to use group LASSO to try to improve results.

### 10 Cell Dual Chain

**Fig 11:**
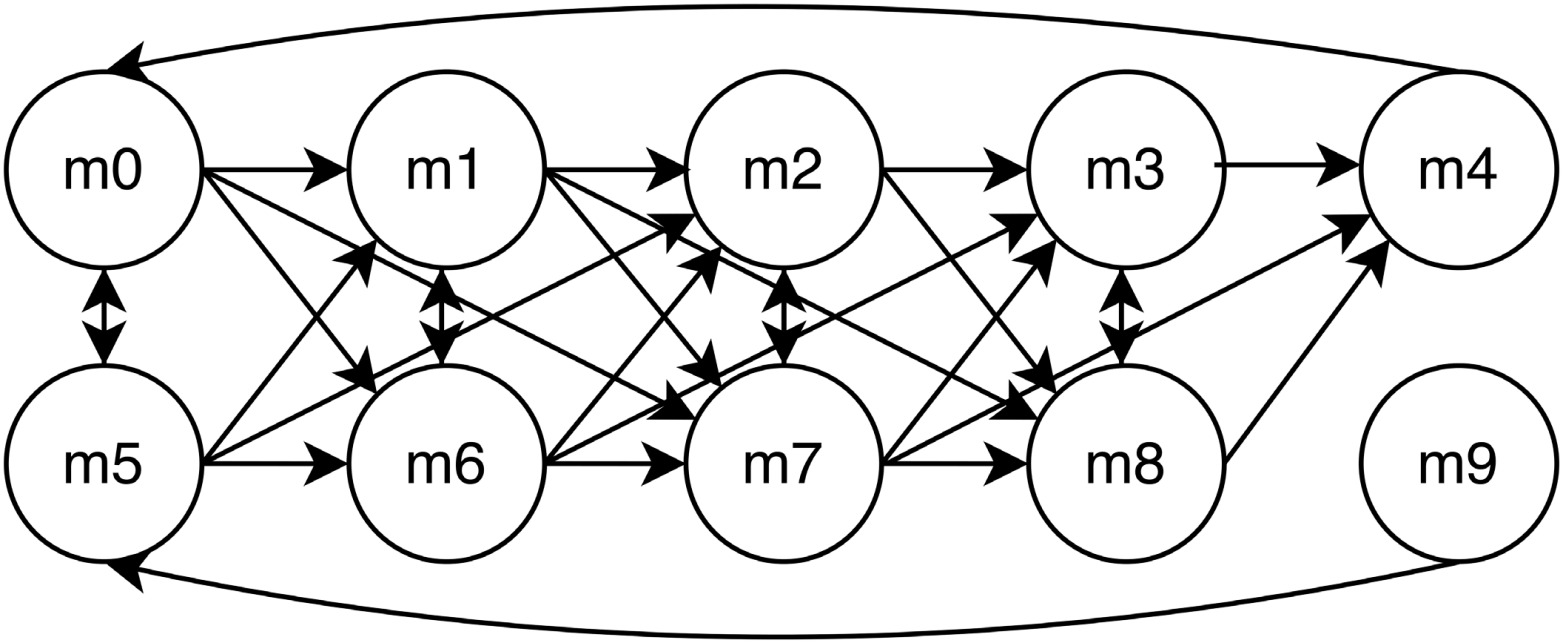
Diagram of 10 Cell Dual Chain Network.

We introduce a 10 Cell Dual Chain cell network depicted in Fig 11, and note the density of its connections as compared to the two previous cell networks.

**Fig 12:**
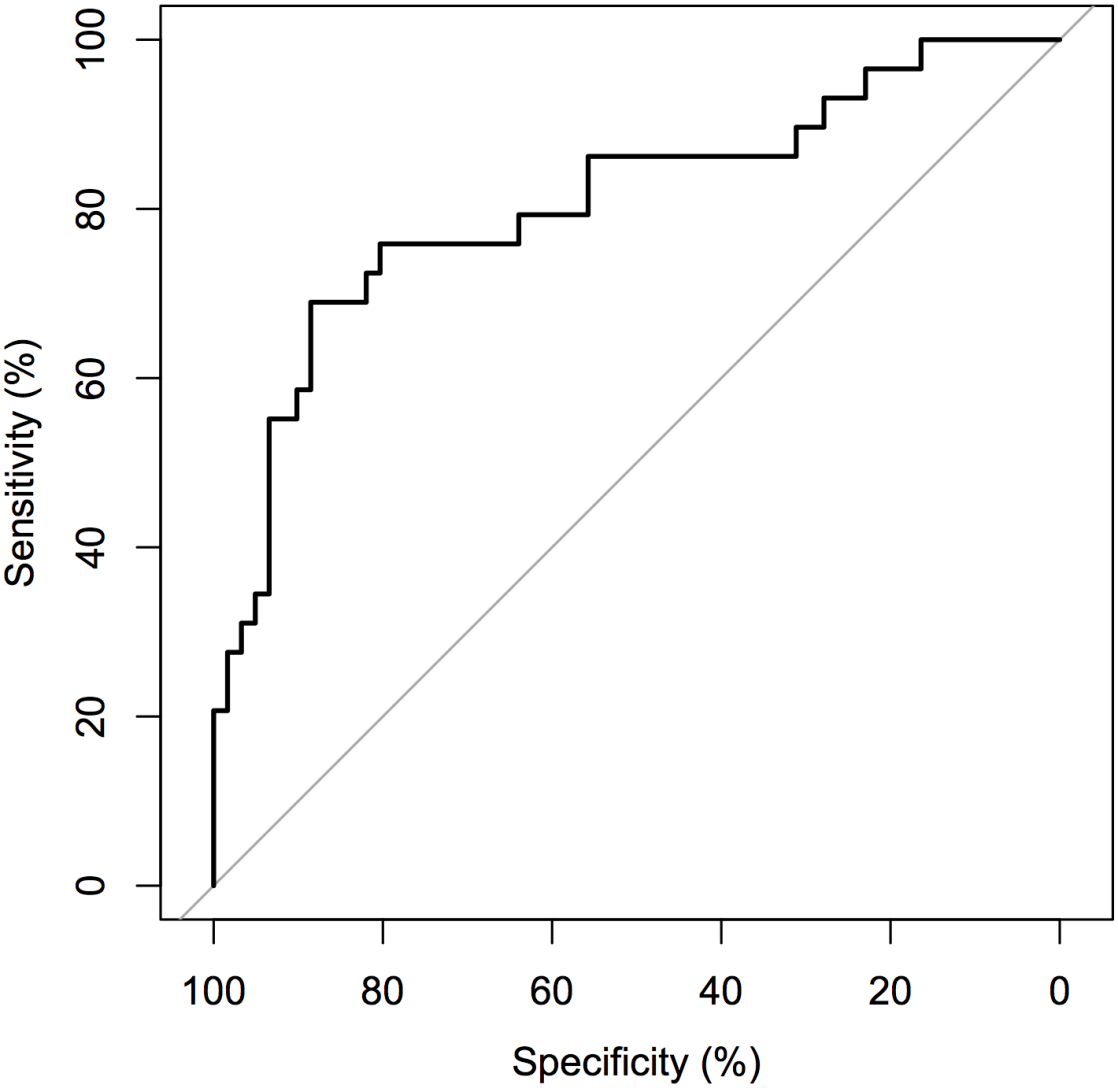
ROC for 10 Cell Network calculated over five hours of observations sampled once a minute. AUC = 80.72%. These five hours of observations were obtained from a subinterval ofa single circadian cycle where PER mRNA is increasing. More observations could be collected and used to improve accuracy from other circadian cycles of the same set of cell traces.

Rather than examining a table of *p*-values, which can be cumbersome, in Fig 12 we display an ROC curve that depicts the accuracy of our inference methodology for observations. The ROC curve has an AUC of 80.72% and is calculated from five hours of observations at one minute intervals.

### 20 Cell Miniature SCN

**Fig 13:**
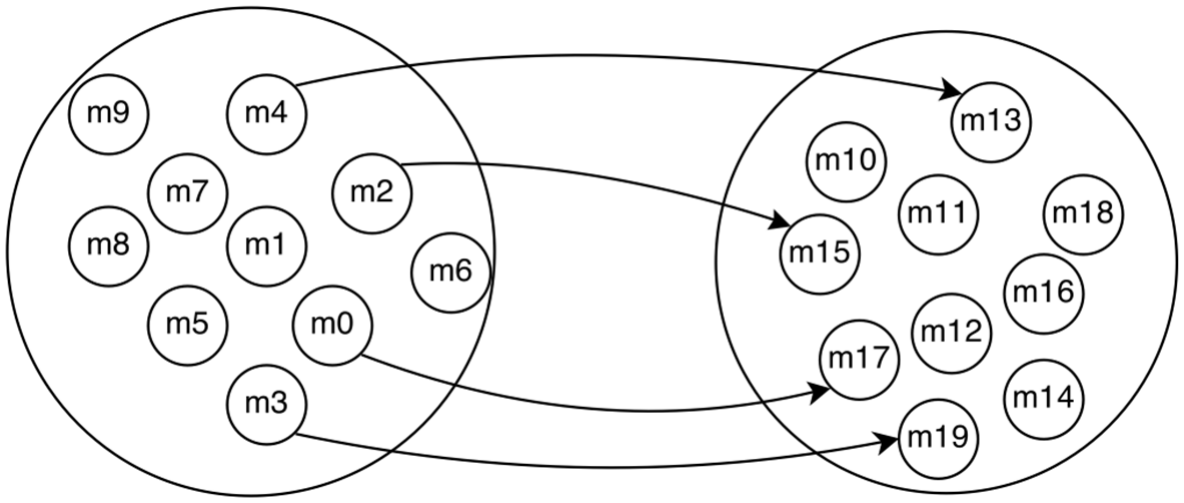
Diagram of 20 Cell Model.

We introduce a twenty cell model that includes two groups of cells that are all connected to each other and four randomly selected cells from one group that influence four randomly selected cells from another group. A diagram of the model is shown in Fig 13.

**Fig 14:**
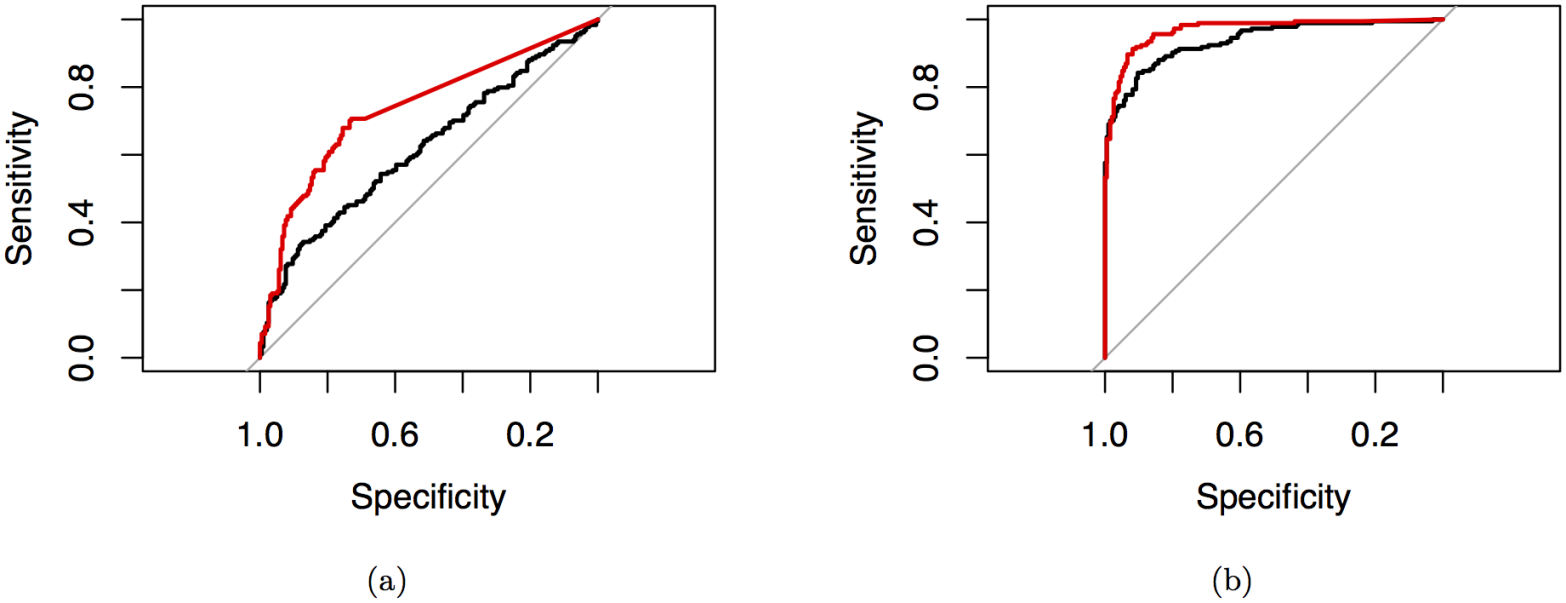
ROC’s of regular Granger causality (black line) and grouped Granger (red line). Fig 12a shows the curves for 14 hours of data sampled every minute. Using group LASSO improved the AUC from 61.99% to 73.78%. Fig 12b shows the curves for 66 hours of data sampled every minute. Using group LASSO improved the AUC from 93.75% to 96.86%.

Fig 14 shows ROC curves calculated from simulated data from the 20 cell miniature SCN model. Obviously, when more observations are used, the inference power is increased, but we include the ROC when fewer observations are used to show how Grouped Granger is most useful when the number of observations is small compared to the number of network connections to be inferred.

### 100 Cell Scale Free and 100 Cell Small World Network

Lastly, we simulate a 100 cell scale free network created using the Barabasi-Albert model [33]. The network has an average node degree of one and is similar in magnitude to true SCN slices. Node degree here was in terms of outgoing connections. We found that for 60 hours of observations sampled every minute our LASSO AFGC method was able to achieve an AUC of 66.07%.

We also generated a 100 cell small world network using the Watts-Strogatz *β* small world algorithm. We used a *beta* = 0.1 and had a network with an average node degree of two. For this network, our algorithm method was able to achieve an AUC of 81.23%.

### AFGC Sensitivity to High Frequency Sampling

**Fig 15:**
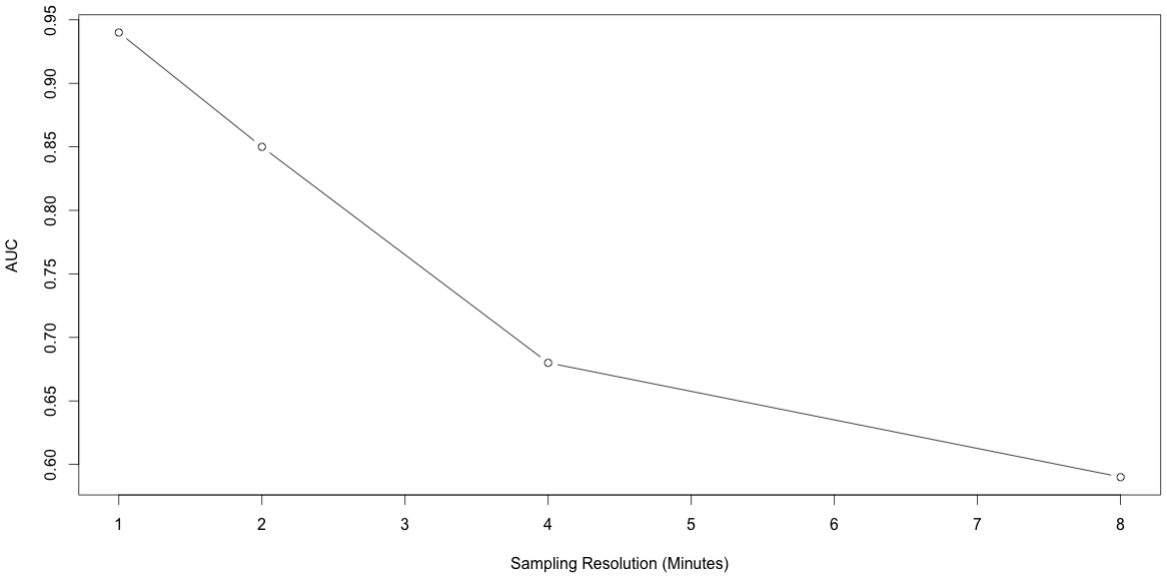
Relationship between AUC and frequency of sampling for AFGC on 10 cell dual chain model. Data was from the same simulation, and number of observations was controlled for by adding twice the number of observations when the sampling rate was doubled.

Because AFGC is a Granger Causality based approach, it is useful for data collected at high frequencies. Although in our results we sampled our systems every minute, we tried our method on lower frequency data of the 10 cell dual chain model to better characterize its effectiveness at lower sampling rates. Fig 15 shows these results. With a sampling rate of one minute, AGFC achieved an AUC of 94.51% for 15 hours of observations. This dropped to an AUC 57.18% of when the system was sampled every eight minutes, controlling for the number of observations by using 120 hours.

For optimal results on SCN recordings, we suggest applying AFGC only when samples are taken at least once every five minutes and ideally once every minute. Applying AFGC to current experimental data, which is typically sampled once every thirty minutes to an hour, will most likely lead to erroneous results. If and when the sampling rate of experimental results reaches five minutes or faster we anticipate AFGC will provide accurate network inference results. As AFGC uses only a portion of the time series for each day, we also recommend applying AFGC to longer recordings. Two weeks of recordings is plenty of recording time for most cases, even when only five hours of recording time is used per day. This is assuming a sampling rate of at least five minutes.

In general, for optimal results the sampling frequency can vary. We suggest looking at cross-correlation plots and validating AFGC on simulated data to see where the cut-off for high frequency noise is for each particular application.

### AFGC Robustness to Measurement Noise

**Fig 16:**
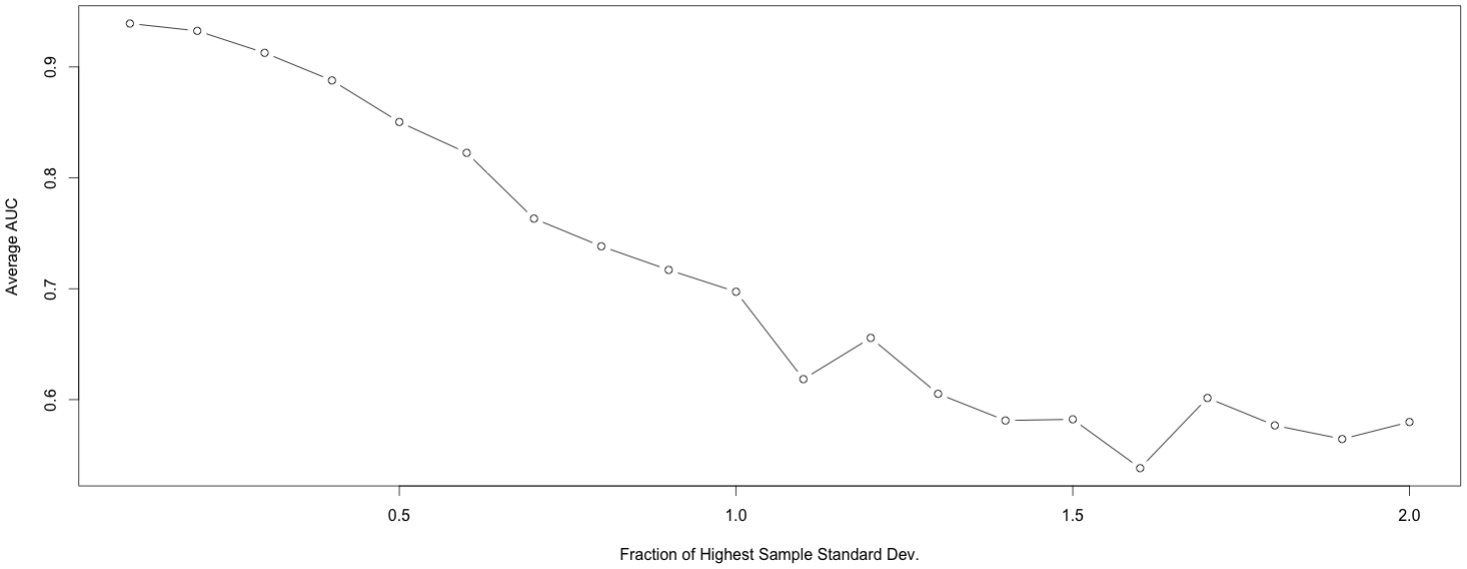
AUC of AFGC when varying levels of noise are added to data from the 10 cell dual chain model. Noise is Gaussian distributed and is characterized by a standard deviation that is a fraction of the highest sample standard deviation of time series of all 10 cells. That is a 1.0 translates to a signal to noise ratio of one. For each level of noise 10 different realizations of the noise was added to the observations, then the AUC was calculated each time and averaged to account for variation in AFGC’s performance from the randomness of the noise.

As experimental recordings often contain measurement noise or are able to record only proxies for mRNA, such as PER2::LUC, we characterized our method’s robustness to measurement noise by running the method on data generated from our 10 cell dual chain model with varying levels of noise. Fig 16 characterizes AFGC’s effectiveness under varying levels of noise.

### A Note on AFGC Applied to System Proxies

In our experiments, all results were obtained by applying AFGC to time series of PER mRNA. This is significant because all coupling between cells in our model is facilitated through the respective PER mRNA levels of each cell. In our model, PER protein in the cytoplasm is a proxy for PER mRNA in a sense, because rising levels of PER mRNA lead to rising levels of PER protein. Thus we would expect these two time series to be approximately correlated in the same cell. This however does not imply that AFGC can recover networks from PER protein data when coupling is exclusively facilitated through PER mRNA. The reasoning is that for our particular model, high frequency noise is damped in the relationship between PER protein and PER mRNA, thus information crucial to AFGC is lost. More specifically, noise in transcription is damped during translation. Noise in the protein arises mostly from the translation process itself. This noise explains why in Fig 2 there is more high frequency noise in the protein trace than in the mRNA trace. For simulations of our 10 cell network we were unable to achieve network inference that was better than random when using the time series of PER protein rather than PER mRNA.

In general, the usefulness of a proxy will depend on how much incoming high frequency noise the proxy filters out. This is highly model dependent. Of course, we expect that in real world scenarios coupling happens through multiple channels and is highly complex. We have shown that AFGC can be highly successful in network inference when applied to signals that are directly responsible for coupling.

### A Note on Applying AFGC When Large Phase Differences Are Present

For all results, we selected portions of the synchronous time series where PER mRNA was rising. These portions were usually at least three hours in length, but often up to seven. Time series need not be synchronous in order for AFGC to work. As long as the linear rising portion of the oscillations match for at least some significant time chunk, AFGC will provide fruitful results. The AUC of 80.72% obtained on the 10 cell network was obtained using only five hours of observations. Thus even if the oscillations contained only one hour of overlapping rising portions, it would only take five days to obtain enough data to conduct that inference.

For larger networks, such as the 100 cell small world network we simulated, lack of synchrony can be more limiting because more sampling is needed and thus recording must go on for longer. In the case of the small world network, 60 hours of observations were needed to obtain an AUC of 81.23%. This would amount to 60 days of observations if only one hour is extractable every day, which unfortunately rules out many experimental recordings. In cases where there are groups of oscillations that exhibit large phase differences from other groups of oscillations or they are not in synchrony at all with other oscillations, we recommend conducting AFGC separately on the different groups. In these cases we can at least extract network topology within the group and assume weak coupling between groups.

## Discussion

Granger Causality has proven to be an effective method for detecting direct causality in multivariate time series but is applicable only when data meets certain assumptions. These assumptions include, but are not limited, to linearity of the system, normality of noise, and stationarity of time series. Furthermore, the number of observations in the time series must be large relative to the number of cells in a system.

We have proposed a methodology for application of Granger Causality to circadian data, for the detection of functional networks. This technique is able to accommodate the assumptions that are required by Granger Causality through the use of approximation and differencing techniques. The technique works by first selecting a specific subsection of each cycle from each of the oscillatory time series. The subsections are then differenced and spliced to form stationary processes of equal lengths. Vector autoregressive models are then fit to these stationary time series and tests are conducted on parameters to assess Granger Causality and answer the question at hand.

We also showed a way to improve the results of this technique when the number of time course observations are small relative to the number of cells in a system. This involves penalizing parameter estimates in accordance to the group LASSO methodology. The high level of accuracy that was displayed by our method on simulated circadian networks provides encouraging evidence that one way relationships between circadian cells in the SCN can be detected from time course gene expression data.

**Fig 17:**
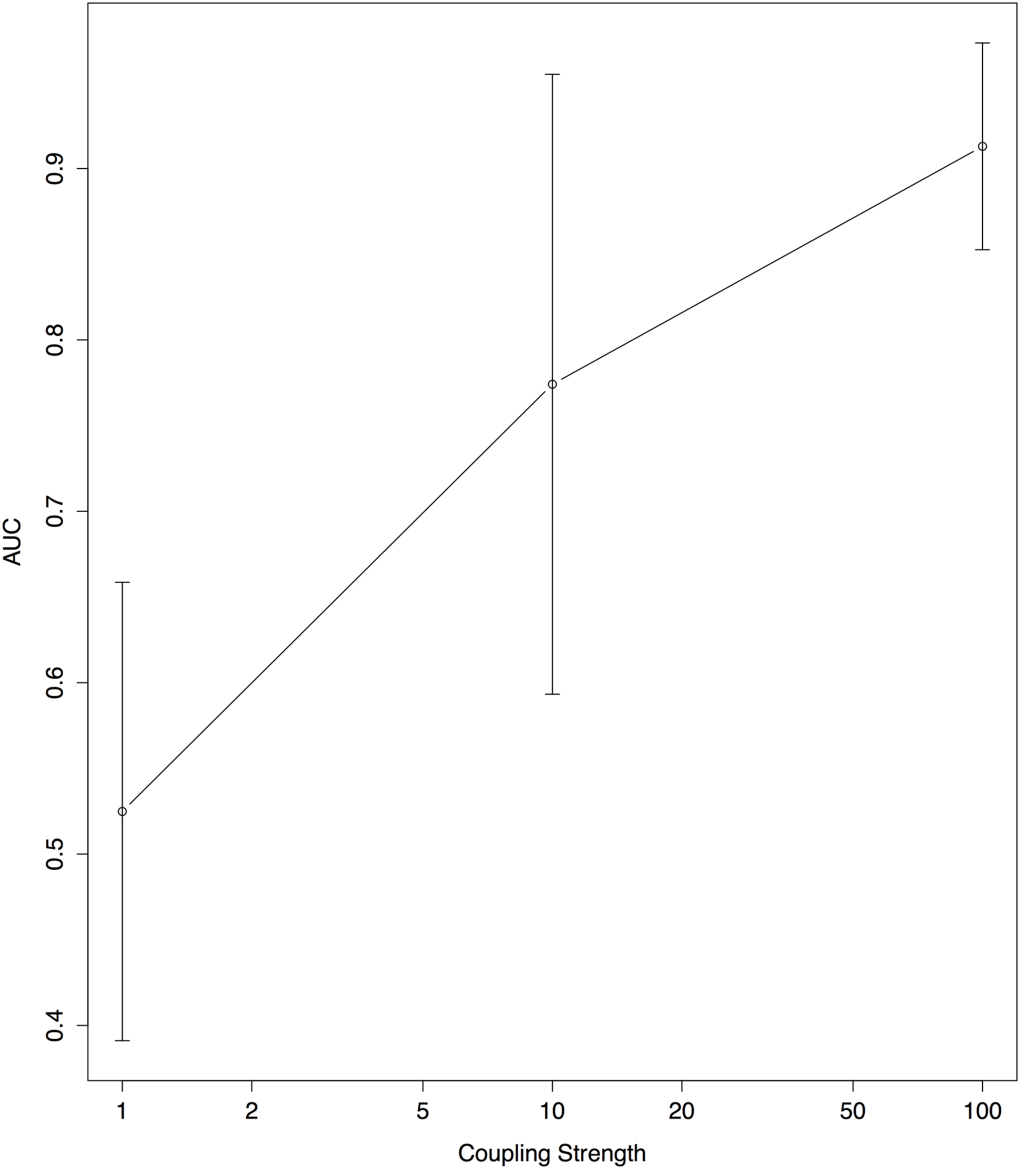
AUC of AFGC on 10 cell dual chain model as coupling strength in the model is varied. Five realizations were run for all three levels of coupling strengths then AFGC was applied to 15 hours of observations for each realization to get an AUC. From these a mean and error was calculated. We note that here coupling strength is on a log scale.

Although our analysis provides an accurate way of detecting direct one way connections between cells in simulated data, it remains to be seen how the method performs on large sets of real biological data. Granger Causality relies on analysis of noise propagation, thus it is a necessary assumption that high frequency noise in cell traces indeed propagates between cells that are connected. We chose a coupling parameter in our model to ensure propagation of noise in simulations given the coupling mechanism. This was to illustrate how well Granger Causality is able to achieve the task of network inference when the propagation exists. Fig 17 formally quantifies how the results of AFGC can change when coupling strength is varied for the 10 cell dual chain model.

Although the mathematical justification of our method relied on the particular form of the coupling mechanism, our method will work under any coupling mechanism that allows for high frequency noise propagation. It is in fact unknown whether high frequency noise propagates between cell traces. We also note that because AFGC is solely reliant on noise propagation, it is robust to minor phase differences between cells. Phases must not be aligned exactly but only so that their upswing in mRNA coincides for some portion of time, since that is when noise best propagates.
